# From Safety Culture Assessment to Organisational Priorities: A Multicentre Study in Surgical Departments

**DOI:** 10.1155/jonm/1939042

**Published:** 2026-11-03

**Authors:** María Virtudes Navarro-García, Manuel Luque-Oliveros, Rubén Morilla-Romero-De-La-Osa

**Affiliations:** ^1^ Department of Nursing Faculty of Nursing Physiotherapy and Podiatry University of Seville Seville Spain us.es; ^2^ Clinical Management Unit of Anesthesiology and Recovery Virgen Del Rocío University Hospital Seville Spain huvr.es; ^3^ Cardiovascular and Thoracic Surgery Operating Theatre Unit Virgen Macarena University Hospital Seville Spain hospital-macarena.com; ^4^ Institute of Biomedicine of Seville (IBiS) Virgen Del Rocío University Hospital/CSIC/University of Seville Seville Spain; ^5^ Biomedical Research Networking Center for Epidemiology and Public Health (CIBERESP) Seville Spain

**Keywords:** operating rooms, organisational culture, patient safety, safety management

## Abstract

**Aim(s):**

To assess patient safety culture in surgical departments, identify factors associated with overall patient safety grade and incident reporting and examine implications for nursing management.

**Background:**

Evidence on factors associated with the principal outcomes of patient safety culture remains limited.

**Method(s):**

A multicentre cross‐sectional study was conducted in surgical departments of three Spanish university hospitals. Hierarchical logistic regression examined factors associated with high overall patient safety grade and incident reporting.

**Results:**

Of 1340 eligible healthcare professionals, 336 participated (25.1%). Teamwork Within Units was the only strength. Organisational Learning, Communication Openness, Management Support and Teamwork Across Units were independently associated with high overall patient safety grade, with the largest association for Teamwork Across Units (aOR 12.93, 95% CI 3.31–54.67, *p* < 0.001). Professional experience was associated with incident reporting (aOR 1.03, 95% CI 1.01–1.06, *p* = 0.009), whereas Organisational Learning showed an inverse association (aOR 0.39, 95% CI 0.15–0.97, *p* = 0.045).

**Conclusion(s):**

Overall patient safety grade and incident reporting are complementary but distinct indicators of patient safety culture.

**Implications for Nursing Management:**

Our findings may help nurse leaders identify potential areas for improvement and distinguish strategies addressing safety perceptions from those addressing incident reporting.

## 1. Introduction

Patient safety is a fundamental component of healthcare quality and is defined by the World Health Organization (WHO) as the absence of preventable harm during healthcare and the reduction of unnecessary risk to an acceptable minimum [1]. This concept encompasses the prevention of adverse events and the minimisation of healthcare errors, considering current knowledge, available resources and the context in which care is delivered. Ensuring patient safety has become a central priority for patients, healthcare professionals and healthcare organisations alike, as it underpins trust in healthcare delivery and the pursuit of safe, effective and high‐quality clinical practice [2].

Consequently, healthcare organisations have developed systematic strategies to evaluate and strengthen patient safety culture, understood as the shared values, beliefs, attitudes and behaviours that determine how safety is embedded within everyday organisational practice [1, 3]. A positive safety culture promotes open communication, organisational learning from errors, teamwork and a system‐based approach to risk management. Regular assessment of patient safety culture has been recommended as a strategy to identify organisational strengths and weaknesses and guide patient safety improvement initiatives [4, 5].

In surgical settings, patient safety culture assumes particular importance because surgical care involves multidisciplinary teamwork, complex workflows and multiple high‐risk processes requiring effective communication and coordination among professionals [6]. International initiatives such as the WHO’s *Safe Surgery Saves Lives* programme have demonstrated the value of structured interventions, including surgical safety checklists, to reduce preventable harm. Nevertheless, the effectiveness of these interventions depends largely on the organisational culture in which they are implemented, making the identification of organisational strengths and barriers essential for achieving sustainable improvements in surgical safety [7, 8].

Patient safety culture has been extensively evaluated using the Hospital Survey on Patient Safety Culture (HSOPSC) across different healthcare settings, including surgical departments. Most studies have focused primarily on describing safety culture profiles, identifying strengths and weaknesses across their dimensions or comparing perceptions between professional groups or institutions [5, 9, 10]. By contrast, relatively few studies have examined how the questionnaire’s safety climate outcome measures relate simultaneously to organisational dimensions of patient safety culture and to professionals’ individual or occupational characteristics, particularly within multicentre surgical settings [6, 11, 12]. Better understanding these relationships may help identify organisational factors associated with safety perceptions and reporting behaviour and inform future quality‐improvement initiatives in surgical practice.

Beyond its multidimensional assessment of patient safety culture, the HSOPSC also incorporates several safety climate outcome measures. In the present study, we focused on two of these measures: the overall patient safety grade and the number of events reported during the previous year. According to the original instrument and its validated Spanish adaptation, these measures complement the multidimensional assessment of patient safety culture by reflecting professionals’ overall appraisal of safety and a key safety‐related behaviour, namely incident reporting. Although they do not represent the multidimensional construct of patient safety culture by themselves, analysing them alongside the organisational dimensions of the HSOPSC may provide a more comprehensive understanding of the factors associated with patient safety culture in surgical departments [13].

To address this gap, the present study was conducted in three large tertiary university hospitals in southern Spain, characterised by high surgical volume, multidisciplinary operating room teams and an important role in undergraduate and postgraduate healthcare education. Before this study, institutional data suggested a relatively low number of formally reported adverse events in surgical departments despite the inherent complexity and risk profile of these settings. Although reporting frequency cannot be interpreted as a direct measure of patient safety performance, it may reflect important aspects of organisational culture, including professionals’ willingness to report incidents, organisational learning and openness of communication. Previous institutional reviews and professional feedback also identified recurrent concerns related to communication openness, feedback about errors, staffing and nonpunitive response to errors, dimensions that have consistently been identified in the international literature as among the principal weaknesses of patient safety culture in surgical environments [4, 11, 12, 14, 15]. Assessing patient safety culture in these hospitals was therefore considered an essential first step to establish a baseline for future quality improvement initiatives and to identify potential organisational areas for further evaluation in surgical care.

Accordingly, the primary objective of this study was to describe the patient safety culture profile in surgical departments across three university hospitals in southern Spain using the validated Spanish version of the HSOPSC. As secondary objectives, we explored the factors associated with the questionnaire’s safety climate outcome measures—overall patient safety grade and the number of events reported during the previous year—by examining the contribution of sociodemographic characteristics, occupational factors and organisational dimensions of patient safety culture. Beyond providing a descriptive assessment of patient safety culture, this study sought to identify organisational and occupational factors associated with the study outcomes, thereby generating hypotheses for future quality‐improvement initiatives in surgical departments.

## 2. Methods

### 2.1. Study Design and Setting

A multicentre cross‐sectional study was conducted in the surgical departments of three university hospitals in the same metropolitan area in southern Spain. The setting included operating rooms from different surgical specialties performing scheduled and emergency procedures during morning and afternoon shifts and their postanaesthesia recovery units. The study was conducted and reported in accordance with the STROBE Statement for cross‐sectional studies.

### 2.2. Participants

A multiprofessional sample was included because patient safety culture reflects shared organisational processes within surgical departments rather than the perspective of a single professional group. Eligible participants were physicians, nurses and nursing assistants assigned to the participating surgical departments, including professionals with supervisory or managerial responsibilities who contributed to the organisational patient safety culture. Administrative staff, porters and maintenance personnel were excluded because they were not involved in clinical care or patient safety management. No formal sample size calculation was performed. The survey was offered to all healthcare professionals meeting the eligibility criteria in the participating surgical departments. The final study size therefore reflected the number of eligible professionals who voluntarily completed the questionnaire during the data collection period.

### 2.3. Data Collection

Before data collection, the study protocol was presented to the heads of the participating surgical departments, who authorised implementation of the survey. Data were collected between October 2023 and January 2024 using an anonymous, self‐administered paper questionnaire. Paper questionnaires were personally distributed to eligible healthcare professionals by one of the study authors.

To minimise the influence of temporary staff and seasonal workforce changes, questionnaires were not distributed during the summer vacation period. Participants received written information describing the study objectives and provided written informed consent before completing the questionnaire. Only completed questionnaires deposited by participants in anonymous collection boxes located in each participating department were included in the analysis.

### 2.4. Instrument and Study Variables

Patient safety culture was assessed using the validated Spanish version of the HSOPSC, developed by the Agency for Healthcare Research and Quality and adapted and validated for Spain. The questionnaire comprises 42 items across 12 dimensions and two outcome measures: overall patient safety grade and incident reporting during the previous 12 months.

Negatively worded items were reverse‐coded according to the Spanish validation manual so that higher scores reflected more favourable safety perceptions. Dimension scores were calculated as the proportion of positive responses, ranging from 0 to 1. For descriptive analyses, dimensions were classified as strengths (≥ 75% positive responses), areas for improvement (≥ 50% to < 75%) or weaknesses (< 50%) according to the Spanish HSOPSC guidelines. Classification was based on the calculated percentages without rounding.

The primary outcomes were overall patient safety grade (item 43), originally measured on a 0–10 scale and dichotomised as high (8–10; event = 1) versus lower (0–7; event = 0) and incident reporting (item 48), originally measured as the number of incidents reported during the previous 12 months and dichotomised as at least one incident reported (event = 1) versus none (event = 0).

### 2.5. Statistical Analysis

Analyses were performed using *R* version 4.4.2. Categorical variables were summarised as frequencies and percentages and continuous variables using appropriate measures of central tendency and dispersion. Group comparisons used Pearson’s *χ* ^2^ or Fisher’s exact tests for categorical variables and parametric or nonparametric tests for continuous variables, as appropriate; continuous associations were assessed using Spearman’s correlation.

Missing data were not imputed, and multivariable analyses used complete cases. Correlations among explanatory variables and variance inflation factors were examined to assess multicollinearity.

Two hierarchical logistic regression models were estimated for each outcome. Model 1 included sex, professional category, age and years of professional experience. Model 2 additionally included the 10 organisational HSOPSC dimensions (D3–D12) to assess their incremental contribution. D1 and D2 were excluded as explanatory variables because they represent HSOPSC outcome measures. Variables were selected a priori based on theoretical and epidemiological relevance, independently of bivariate statistical significance; years working at the hospital and within the department were excluded because of conceptual overlap with professional experience.

Model performance was assessed using likelihood ratio tests, Akaike’s Information Criterion, McFadden’s and Nagelkerke’s pseudo‐R^2^, AUC and the Hosmer–Lemeshow test. Results are reported as adjusted odds ratios with 95% confidence intervals; *p* < 0.05 was considered statistically significant.

For model robustness assessment, the fully adjusted models included 15 predictor parameters, with 170 and 94 outcome events for safety grade and incident reporting, respectively (11.3 and 6.3 events per parameter).

### 2.6. Ethical Considerations

The study protocol was approved by the Research Ethics Committee (CEI) of Virgen Macarena and Virgen del Rocío University Hospitals, Seville, Spain (approval number C.I.0122‐N‐22). Participation was voluntary and anonymous, and written informed consent was obtained from all participants. The study was conducted in accordance with the principles of the Declaration of Helsinki, Regulation (EU) 2016/679 (General Data Protection Regulation), and Spanish Organic Law 3/2018 on the Protection of Personal Data and Guarantee of Digital Rights.

## 3. Results

Of the 1340 eligible healthcare professionals invited to participate, 336 completed the survey, resulting in a response rate of 25.1%. Response rates differed significantly across professional categories (*p* < 0.001; Cramér’s V = 0.147), being higher among nurses (32.2%) than among nursing assistants (18.6%) and physicians (20.4%). Pairwise comparisons confirmed higher response rates among nurses than nursing assistants and physicians (both *p* < 0.001), whereas nursing assistants and physicians did not differ (*p* = 0.560).

The characteristics of the study population are summarised in Table 1. Overall, 249 (74.1%) participants were women, approximately two‐thirds were younger than 50 years, and nurses represented the largest professional group (*n* = 203, 60.8%), followed by nursing assistants (*n* = 70, 21.0%) and physicians (*n* = 61, 18.3%). Most respondents (88%) reported direct contact with patients.

**TABLE 1:** Sample characteristics of surgical department healthcare professionals: Descriptive analysis (*N* = 336).

Variable		*n*	%	95% CI
Age (years)	Less than 30	59	17.8	13.7–21.9
	31–40	63	19	14.8–23.3
	41–50	97	29.3	24.4–34.2
	51–60	77	23.3	18.7–27.8
	> 60	35	10.6	7.3–13.9

Hospital	HA	63	19.1	14.9–23.3
	HB	259	78.5	74.1–82.9
	HC	8	2.4	0.8–4.1

Hours worked per week	Less than 20 h	22	7.1	4.3–10.0
	20–30 h	40	13	9.2–16.7
	31–40 h	203	65.9	60.6–71.2
	More than 40 h	43	14	10.1–17.8

Incidents reported	0	236	70.7	65.8–75.5
	1	25	7.5	4.7–10.3
	2	27	8.1	5.2–11.0
	3	17	5.1	2.7–7.4
	4	4	1.2	0.0–2.4
	5+	25	7.5	4.7–10.3

	**Mean (sd)**	**P50 [P25–75]**	**Range**	**Normal distribution**

Work experience (years)	18.1 (11.6)	18 [8–26]	1–46	No
Work experience in the hospital	13.8 (11.0)	10 [4–20]	1–46	No
Work experience in the department	9.7 (8.4)	7 [3–14]	1–44	No

*Note: p*: percentile.

Abbreviations: 95% CI = confidence interval 95%, sd = standard deviation.

Professional category differed by sex (*p* < 0.001) and by years of professional experience (*p* = 0.004); no other significant differences were observed across these demographic comparisons.

Healthcare professionals reported a generally high overall patient safety grade, with no significant differences according to hospital, professional category, sex or age group.

Regarding incident reporting during the previous 12 months, 70.7% of professionals reported no incidents, whereas 29.3% reported at least one event.

The overall patient safety culture profile is presented in Table 2. Teamwork Within Units was the only dimension classified as a strength (≥ 75% positive responses). Supervisor/Manager Expectations and Actions Promoting Patient Safety and Organisational Learning‐Continuous Improvement were classified as areas for improvement (≥ 50% to < 75% positive responses), whereas the remaining dimensions were classified as weaknesses (< 50% positive responses).

**TABLE 2:** Analysis of strengths and weaknesses in patient safety culture among surgical department healthcare professionals: descriptive analysis (*N* = 336).

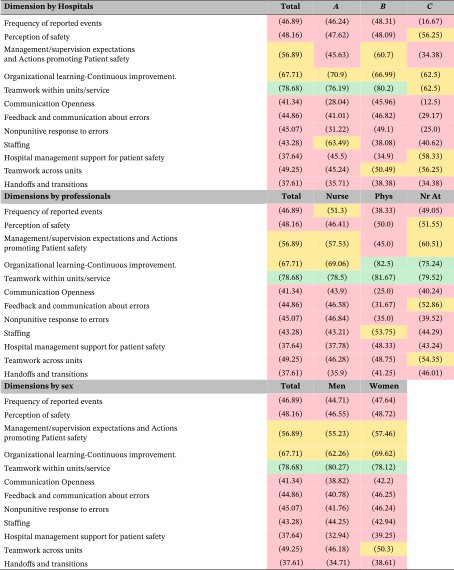

This overall pattern was remarkably consistent across hospitals, professional categories and sex. No statistically significant differences were observed in the distribution of strengths, areas for improvement and weaknesses between hospitals (*p* = 0.766), professional groups (*p* = 0.755) or sex (*p* = 0.985).

The descriptive dimension scores supported this classification, with Teamwork Within Units showing the highest positive score, whereas Hospital Management Support for Patient Safety, Handoffs and Transitions, Communication Openness, Staffing, Feedback and Communication about Error and Nonpunitive Response to Error showed the lowest positive response scores.

Figure 1 illustrates the distribution of negative responses across the HSOPSC dimensions for the overall sample and by professional group. Communication Openness, Nonpunitive Response to Error, Staffing and Hospital Management Support for Patient Safety consistently accounted for the largest proportion of negative responses. Similar patterns were observed across hospitals, professional categories and sex, indicating that the same organisational dimensions concentrated most negative perceptions regardless of subgroup.

**FIGURE 1:**
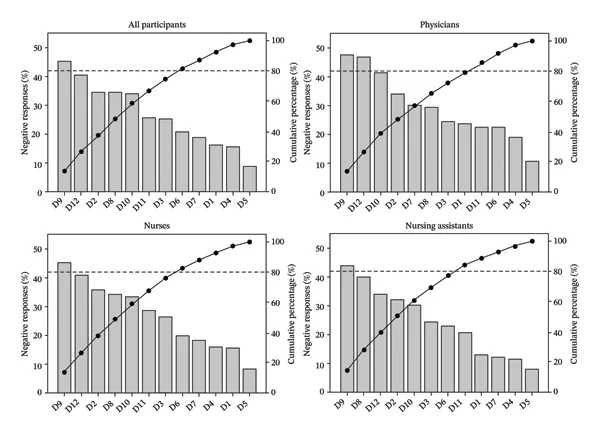
Pareto diagram of negative responses across the 12 patient safety culture dimensions among surgical department healthcare professionals (*N* = 336). Bars represent the percentage of negative responses for each dimension, ranked from highest to lowest; the line represents the cumulative percentage of negative responses. D1, Frequency of Events Reported; D2, Overall Perceptions of Patient Safety; D3, Supervisor/Manager Expectations and Actions Promoting Patient Safety; D4, Organisational Learning‐Continuous Improvement; D5, Teamwork Within Units; D6, Communication Openness; D7, Feedback and Communication About Error; D8, Nonpunitive Response to Error; D9, Staffing; D10, Management Support for Patient Safety; D11, Teamwork Across Units; D12, Handoffs and Transitions.

Bivariate analyses showed differences in several organisational dimensions according to overall patient safety grade (all *p* < 0.05), whereas incident reporting was associated with professional experience (*p* < 0.001), Supervisor/Manager Expectations (*p* = 0.026) and Organisational Learning (*p* = 0.013) (Table 3). Neither outcome differed significantly by sex or professional category.

**TABLE 3:** Bivariate analysis of overall safety grade and incident reporting among surgical department healthcare professionals using Pearson’s *χ* ^2^ and Mann–Whitney *U* tests (*N* = 249 and *N* = 299, respectively).

Variable	Safety grade		Incident reporting	
	Grades 0–7 (*n* = 79)	Grades 8–10 (*n* = 170)	No report (*n* = 205)	≥ 1 report (*n* = 94)
Sex (women)	55 (69.6%)	129 (75.9%)^a^	157 (76.6%)	63 (67.0%)^a^
Profession: nurse assistant	12 (15.2%)	37 (21.8%)	45 (22.0%)	15 (16.0%)
Nurse	51 (64.6%)	104 (61.2%)	122 (59.5%)	59 (62.8%)
Physician	16 (20.3%)	29 (17.1%)^a^	38 (18.5%)	20 (21.3%)^a^
Age, years	45 (34–55)	45 (35–55)^b^	44 (35–55)	45 (37.25–55)^b^
Prof exp, years	19 (10.5–26)	17 (6–26)^b^	16 (6–24)	**22 (15–28)** ^b^ ^∗^
D3	0.50 (0.25–0.50)	**0.50 (0.50–1.00)** ^b^ ^∗^	0.50 (0.50–1.00)	**0.50 (0.25–0.69)** ^b^ ^∗^
D4	0.50 (0–0.67)	**1.00 (0.67–1.00)** ^b^ ^∗^	0.67 (0.33–1.00)	**0.67 (0.33–1.00)** ^b∗^
D5	0.67 (0.42–1.00)	**1.00 (0.67–1.00)** ^b^ ^∗^	1.00 (0.67–1.00)	1.00 (0.67–1.00)^b^
D6	0.33 (0–0.33)	**0.33 (0.33–0.67)** ^b^ ^∗^	0.33 (0–0.67)	0.33 (0–0.67)^b^
D7	0.33 (0–0.67)	**0.67 (0.08–1.00)** ^b^ ^∗^	0.33 (0–0.67)	0.33 (0–0.67)^b^
D8	0.33 (0–0.67)	0.33 (0–0.67)^b^	0.33 (0.33–0.67)	0.33 (0–0.67)^b^
D9	0.50 (0–0.75)	0.50 (0.25–0.75)^b^	0.25 (0–0.75)	0.50 (0.25–0.75)^b^
D10	0.33 (0–0.33)	**0.33 (0–0.67)** ^b^ ^∗^	0.33 (0–0.67)	0.33 (0–0.67)^b^
D11	0.25 (0–0.50)	**0.50 (0.50–0.75)** ^b^ ^∗^	0.50 (0.25–0.75)	0.50 (0.25–0.69)^b^
D12	0.25 (0.25–0.50)	0.25 (0.25–0.50)^b^	0.50 (0–0.75)	0.25 (0.25–0.50)^b^

*Note:* Values are *n* (%) for categorical variables and median (IQR) for continuous variables. D3, Supervisor/Manager Expectations and Actions Promoting Patient Safety; D4, Organisational Learning‐Continuous Improvement; D5, Teamwork Within Units; D6, Communication Openness; D7, Feedback and Communication About Error; D8, Nonpunitive Response to Error; D9, Staffing; D10, Management Support for Patient Safety; D11, Teamwork Across Units; D12, Handoffs and Transitions. Bivariate analyses showed that the overall patient safety grade was significantly associated with Supervisor/Manager Expectations and Actions (D3; *p* < 0.001), Organizational Learning—Continuous Improvement (D4; *p* < 0.001), Teamwork Within Units (D5; p = 0.009), Communication Openness (D6; *p* < 0.001), Feedback and Communication About Error (D7; *p* < 0.001), Management Support for Patient Safety (D10; *p* < 0.001), and Teamwork Across Units (D11; *p* < 0.001). Incident reporting was significantly associated with professional experience (*p* < 0.001), Supervisor/Manager Expectations and Actions (D3; *p* = 0.026), and Organizational Learning—Continuous Improvement (D4; *p* = 0.013).

^a^Pearson’s *χ* ^2^ test.

^b^Mann–Whitney *U* test.

^∗^ *p* < 0.05.

The hierarchical multivariable models for a high overall patient safety grade are presented in Table 4. After adjustment, Organisational Learning (aOR 4.00, 95% CI 1.40–11.83, *p* = 0.011), Communication Openness (aOR 3.97, 95% CI 1.13–14.71, *p* = 0.034), Management Support (aOR 3.72, 95% CI 1.11–13.21, *p* = 0.037) and Teamwork Across Units (aOR 12.93, 95% CI 3.31–54.67, *p* < 0.001) remained independently associated with a high overall patient safety grade. Adding the organisational dimensions significantly improved model fit (likelihood ratio test, *p* < 0.001) and discrimination (AUC: 0.575–0.813).

**TABLE 4:** Hierarchical logistic regression of high overall patient safety grade among surgical department healthcare professionals (*N* = 249).

**Block**	**Variable**	**Model 1 adj OR (95% CI)**	**p**	**Model 2 adj OR (95% CI)**	**p**

Individual characteristics	Sex (male vs. female)	1.25 (0.68–2.29)	0.467	1.42 (0.68–2.95)	0.348
	Profession (physician vs. nurse)	0.83 (0.41–1.72)	0.606	1.02 (0.42–2.51)	0.962
	Profession (nursing assistant vs. nurse)	1.43 (0.68–3.18)	0.358	1.57 (0.64–4.00)	0.334
	Age	1.01 (0.98–1.04)	0.458	0.99 (0.96–1.02)	0.448
	Prof exp (years)	0.98 (0.95–1.00)	0.060	0.98 (0.95–1.01)	0.289

Organisational dimensions (HSOPSC)	D3: Supervisor/Manager Expectations			2.17 (0.55–8.76)	0.272
	D4: Organisational Learning			4.00 (1.40–11.83)	0.011
	D5: Teamwork Within Units			1.02 (0.30–3.40)	0.977
	D6: Communication Openness			3.97 (1.13–14.71)	0.034
	D7: Feedback About Errors			0.77 (0.24–2.40)	0.658
	D8: Nonpunitive Response			0.97 (0.35–2.68)	0.945
	D9: Staffing			0.46 (0.17–1.23)	0.123
	D10: Management Support			3.72 (1.11–13.21)	0.037
	D11: Teamwork Across Units			12.93 (3.31–54.67)	< 0.001
	D12: Handoffs and Transitions			0.34 (0.09–1.23)	0.103

**Model performance**	**Model 1**			**Model 2**	

AIC	317.22			267.86	
Likelihood ratio test	—			*χ* ^2^ = 69.36; *p* < 0.001	
McFadden *R* ^2^	0.019			0.242	
Nagelkerke *R* ^2^	0.033			0.366	
AUC	0.575			0.813	
Hosmer–Lemeshow	*p* = 0.546			*p* = 0.129	

*Note:* Adj OR, adjusted odds ratio; 95% CI, 95% confidence interval; Prof exp, years of professional experience; HSOPSC, Hospital Survey on Patient Safety Culture; AUC, area under the receiver operating characteristic curve; D3, Supervisor Expectations and Actions Promoting Patient Safety; D4, Organisational Learning‐Continuous Improvement; D5, Teamwork Within Units; D6, Communication Openness; D7, Feedback and Communication About Error; D8, Nonpunitive Response to Error; D9, Staffing; D10, Hospital Management Support for Patient Safety; D11, Teamwork Across Hospital Units; D12, Handoffs and transitions.

Abbreviation: AIC = Akaike information criterion.

For incident reporting, greater professional experience remained independently associated with higher odds of reporting at least one incident (aOR 1.03, 95% CI 1.01–1.06, *p* = 0.009), whereas Organisational Learning showed an inverse association (aOR 0.39, 95% CI 0.15–0.97, *p* = 0.045) (Table 5). Adding the organisational dimensions did not significantly improve model fit (likelihood ratio test, *p* = 0.104).

**TABLE 5:** Hierarchical logistic regression of incident reporting among surgical department healthcare professionals (*N* = 299).

**Variable**	**Model 1 adj OR (95% CI)**	***p***	**Model 2 adj OR (95% CI)**	***p***

Sex, male vs female	0.61 (0.35–1.08)	0.091	0.64 (0.36–1.16)	0.138
Physician vs nurse	1.21 (0.62–2.32)	0.575	1.09 (0.53–2.20)	0.820
Nursing assistant vs nurse	0.64 (0.31–1.26)	0.207	0.61 (0.29–1.26)	0.194
Age	1.01 (0.99–1.04)	0.349	1.02 (1.00–1.05)	0.076
Years of professional experience	1.03 (1.01–1.06)	0.009	1.03 (1.01–1.06)	0.009
D3: Supervisor/Manager Expectations	—	—	0.48 (0.16–1.44)	0.189
D4: Organisational Learning	—	—	0.39 (0.15–0.97)	0.045
D5: Teamwork Within Units	—	—	2.21 (0.80–6.43)	0.135
D6: Communication Openness	—	—	0.81 (0.29–2.26)	0.691
D7: Feedback About Errors	—	—	1.14 (0.45–2.89)	0.789
D8: Nonpunitive Response to Error	—	—	0.70 (0.30–1.66)	0.423
D9: Staffing	—	—	1.90 (0.86–4.28)	0.115
D10: Management Support	—	—	0.85 (0.33–2.22)	0.745
D11: Teamwork Across Units	—	—	0.88 (0.29–2.68)	0.827
D12: Handoffs and Transitions	—	—	1.02 (0.34–3.00)	0.971

**Model performance**	**Model 1**		**Model 2**	

AIC	368.79		372.92	
Likelihood ratio test	—		*χ* ^2^ = 15.87; *p* = 0.104	
McFadden *R* ^2^	0.02365		0.06682	
Nagelkerke *R* ^2^	0.04073		0.11204	
AUC	0.5997		0.6641	
Hosmer–Lemeshow	0.3353		0.8321	

*Note:* Adj OR, adjusted odds ratio; D3, Supervisor Expectations and Actions Promoting Patient Safety; D4, Organisational Learning–Continuous Improvement; D5, Teamwork Within Units; D6, Communication Openness; D7, Feedback and Communication About Error; D8, Nonpunitive Response to Error; D9, Staffing; D10, Hospital Management Support for Patient Safety; D11, Teamwork Across Hospital Units; D12, Handoffs and Transitions.

Abbreviations: 95% CI = 95% confidence interval, AIC = Akaike information criterion.

## 4. Discussion

This multicentre study provides a comprehensive assessment of patient safety culture in Spanish surgical departments and extends current evidence by examining the relative contribution of organisational and individual factors to two key HSOPSC outcomes. The findings revealed strong teamwork within clinical units but persistent weaknesses in communication, organisational support and coordination across services. Organisational dimensions substantially improved the model for a high overall patient safety grade but not for incident reporting, for which professional experience remained independently associated. These findings indicate that safety perceptions and reporting behaviours represent complementary but distinct constructs, with implications for interpreting HSOPSC results and designing organisational interventions.

The overall pattern of patient safety culture was consistent with previous reports from surgical settings across healthcare systems. Teamwork Within Units emerged as the strongest organisational attribute, whereas Communication Openness, Nonpunitive Response to Error, Staffing, Management Support for Patient Safety and Teamwork Across Hospital Units were among the weakest domains [5, 11, 16, 17]. The persistence of this pattern across countries suggests that these deficiencies may represent common organisational challenges in complex surgical environments rather than isolated local problems.

Beyond confirming these patterns, only a limited number of organisational dimensions remained independently associated with a high overall patient safety grade after adjustment for individual characteristics. This suggests that organisational domains may differ in their relevance to professionals’ global safety perceptions. When resources are limited, dimensions showing the strongest independent associations may represent candidate areas for further evaluation and may help generate hypotheses for future quality‐improvement interventions.

From a nursing leadership perspective, Organisational Learning‐Continuous Improvement, Communication Openness, Management Support for Patient Safety and Teamwork Across Hospital Units represent organisational domains warranting further evaluation. This is consistent with evidence emphasising organisational processes, alongside individual‐level initiatives, in strengthening patient safety culture [18, 19]. Although specific interventions were not evaluated in this study, strategies aligned with these dimensions include visible leadership, structured multidisciplinary safety discussions, feedback following incident reporting and mechanisms for incident review and organisational learning [19, 20].

Such strategies may foster psychologically safe environments that support speaking up, sharing concerns and continuous safety improvement. Rather than identifying these findings as established intervention priorities, they may help nurse leaders identify organisational conditions that warrant further evaluation, particularly those related to communication, learning and collaboration across professional and departmental boundaries. This perspective conceptualises patient safety culture as an organisational capability rather than an aggregation of individual attitudes and may explain why interventions focused exclusively on individual behaviour can be difficult to sustain or insufficient to transform safety culture.

One of the most relevant findings of this study is that the two principal outcomes included in the HSOPSC behaved differently in the multivariable analyses. Although incorporating the organisational dimensions substantially improved the model for a high overall patient safety grade, these dimensions did not provide significant incremental explanatory value for incident reporting beyond individual characteristics. Moreover, professional experience remained independently associated with reporting behaviour. These findings suggest that, although both outcomes are related to patient safety culture, they capture different aspects of organisational safety and should therefore not be interpreted interchangeably.

Overall patient safety grade represents a global cognitive appraisal of the safety climate within the organisation. It reflects how professionals integrate their day‐to‐day experiences of leadership, communication, teamwork, organisational learning and managerial support into an overall judgement of safety. Accordingly, it is not surprising that organisational dimensions explained a substantial proportion of the variability in this outcome. Similar observations have been reported in previous studies showing that leadership behaviours, organisational support and transformational leadership are among the strongest determinants of professionals’ perceptions of patient safety culture [18, 20, 21].

In contrast, reporting an incident constitutes professional behaviour rather than perception. Before an incident is reported, several conditions must be met: an event must occur or be recognised, the professional must judge it worthy of reporting, understand the reporting procedure, believe that reporting will lead to meaningful improvement, and feel sufficiently supported to report without fear of blame or negative consequences. Consequently, incident reporting depends not only on safety culture but also on reporting systems, organisational justice, psychological safety, workload, leadership behaviours, previous reporting experiences and the availability of effective feedback mechanisms [18, 19].

The independent association between professional experience and incident reporting represents an additional finding of the present study. Greater professional experience was associated with higher odds of having reported at least one incident during the previous year, and this association remained after adjustment for the organisational HSOPSC dimensions. Previous studies have primarily examined incident reporting in relation to organisational dimensions or reporting barriers [4, 11, 15], although recent evidence has also identified professional tenure as associated with reporting behaviour [18]. More experienced professionals may be better able to recognise reportable events, may have greater familiarity with institutional reporting procedures or may feel more confident in initiating a report. Professional experience may also reflect cumulative exposure to clinical situations in which reportable incidents occur. However, these mechanisms were not directly assessed in the present study and should therefore be regarded as hypotheses requiring further investigation.

This interpretation may also help explain an apparently paradoxical pattern observed in our sample and described in previous reports: favourable overall safety perceptions may coexist with relatively low reporting rates [17, 18]. Rather than indicating inconsistency, this pattern highlights that reporting behaviour is influenced by barriers extending beyond professionals’ safety perceptions. Fear of blame, lack of standardised reporting procedures, insufficient feedback following reports, competing workload demands, and limited opportunities for organisational learning have all been identified as barriers that reduce reporting despite otherwise positive safety climates [18, 19].

From a nursing management perspective, incident reporting should not be interpreted as a standalone indicator of patient safety culture. Low reporting does not necessarily imply poor safety culture, nor does high reporting automatically indicate safer organisations. Nurse leaders should therefore interpret reporting alongside communication, leadership support, teamwork and organisational learning. Reporting systems are most effective when embedded within safety cultures characterised by visible leadership, open communication, feedback and learning‐oriented practices [19, 20]. The relationship may also be bidirectional: supportive safety environments facilitate reporting, while effective reporting creates opportunities to identify vulnerabilities and strengthen organisational learning.

More broadly, the value of HSOPSC may extend beyond describing organisational strengths and weaknesses to informing areas for further organisational evaluation. In resource‐constrained surgical environments, targeting domains most strongly associated with relevant safety outcomes may provide candidate areas for subsequent evaluation and quality‐improvement initiatives. This approach is consistent with contemporary models emphasising coordinated organisational processes involving leadership, care practices and staff engagement [22–24].

For nurse leaders, this requires actively shaping the organisational environment through practices such as visible leadership, structured safety conversations, multidisciplinary safety huddles, feedback following incident reporting and systematic review of safety events [20, 23, 25]. Although safety culture is inherently multiprofessional, nurse managers are well positioned to act on several organisational dimensions assessed in this study, particularly teamwork, communication, organisational learning and care coordination. A multiprofessional assessment also provides nurse managers with a broader view of the surgical safety environment, helping to distinguish organisational issues requiring shared interdisciplinary strategies from those that may warrant nursing‐specific interventions. Dimensions such as management support, communication openness and teamwork across hospital units cannot be modified through individual training alone but require alignment between leadership, organisational priorities, communication structures and learning systems. Multilevel approaches involving leadership, care processes and staff engagement may achieve more sustained improvements than isolated interventions [19, 22].

Patient safety culture assessment should therefore be linked to organisational action. Its value lies in informing feedback, learning, leadership decisions and resource allocation. By distinguishing overall safety perceptions from reporting behaviours and identifying organisational domains with stronger independent associations, HSOPSC may provide nurse leaders with a more focused basis for identifying areas requiring further organisational evaluation within surgical services.

### 4.1. Strengths and Limitations

This study has several strengths, including its multicentre design, the inclusion of different professional groups, the use of the validated Spanish HSOPSC with standardised interpretation criteria and a hierarchical modelling strategy that examined the relative contribution of individual and organisational factors to the two principal HSOPSC outcomes.

Several limitations should be considered. The cross‐sectional design precludes causal inference, and self‐reported data may be affected by recall bias, social desirability or local reporting norms. Incident reporting was based on professionals’ self‐reported behaviour during the previous 12 months rather than institutional records. The overall response rate was low (25.1%). Although reasons for nonparticipation were not collected, time constraints, high workload and survey fatigue have been identified as barriers to participation in safety culture surveys among healthcare professionals [26, 27]. Concerns about expressing critical views of organisational safety may also have discouraged participation, although the anonymous nature of the survey was intended to minimise this possibility. Consequently, systematic differences between respondents and nonrespondents cannot be excluded.

Nurses showed higher response rates than nursing assistants and physicians. This may partly reflect greater professional proximity to the nursing‐led research team, including two authors working in participating hospitals, although reasons for nonparticipation were not collected. The resulting overrepresentation of nurses may have increased the contribution of nursing perspectives to the observed safety culture.

The lower event‐to‐parameter ratio for the incident‐reporting model also warrants caution regarding the precision and stability of its estimates.

Finally, the inclusion of surgical departments from three university hospitals within the same metropolitan area and healthcare system limits generalisability. Findings may therefore not be transferable to nonuniversity or rural hospitals or to healthcare systems with different organisational and reporting cultures.

## 5. Conclusions

This study provides a comprehensive assessment of patient safety culture in surgical departments, identifying Teamwork Within Units as an organisational strength and persistent weaknesses in Communication Openness, Management Support for Patient Safety, Staffing and Teamwork Across Hospital Units.

The two principal HSOPSC outcomes captured different aspects of organisational safety. Overall patient safety grade was strongly associated with key organisational domains, whereas organisational dimensions did not significantly improve the explanation of incident reporting beyond individual characteristics, and professional experience remained independently associated with reporting behaviour. Safety perceptions and reporting behaviours should therefore be regarded as complementary rather than interchangeable indicators.

From a nursing management perspective, not all patient safety culture dimensions appear to have the same strategic relevance. Given the cross‐sectional design, these findings represent associations rather than causal relationships; prospective intervention studies are needed to determine whether modifying these dimensions improves patient safety outcomes. Nurse leaders may use the organisational domains most strongly associated with favourable safety perceptions to identify potential priorities for further evaluation, while adopting distinct strategies for incident reporting. Used in this way, patient safety culture assessments can inform leadership decisions, resource allocation and organisational learning.

## Author Contributions

The manuscript has not been submitted elsewhere nor previously published. Study conception and design: María Virtudes Navarro‐García, Manuel Luque‐Oliveros, Rubén Morilla‐Romero‐De‐La‐Osa, Data collection: María Virtudes Navarro‐García, Manuel Luque‐Oliveros. Data analysis and interpretation: María Virtudes Navarro‐García, Rubén Morilla‐Romero‐De‐La‐Osa. Drafting of the article: María Virtudes Navarro‐García, Manuel Luque‐Oliveros. Critical revision of the article: Rubén Morilla‐Romero‐De‐La‐Osa. All authors contributed to the study design, data collection, data analysis and manuscript writing.

## Funding

This study did not receive funding from public, commercial or nonprofit agencies.

## Disclosure

All authors have read and approved the final version of the manuscript.

## Conflicts of Interest

The authors declare no conflicts of interest.

## Supporting Information

Additional supporting information can be found online in the Supporting Information section.

## Supporting information


**Supporting Information** STROBE Checklist.

## Data Availability

The dataset is not publicly available because public data sharing was not included in the participant consent or ethical approval for this study. De‐identified data may be made available by the corresponding author upon reasonable request, subject to applicable ethical and institutional requirements.
